# Sequence analysis of coding and 3' and 5' flanking regions of the epithelial sodium channel α, β, and γ genes in Dahl S *versus *R rats

**DOI:** 10.1186/1471-2156-8-35

**Published:** 2007-06-25

**Authors:** Marlene F Shehata, Frans HH Leenen, Frédérique Tesson

**Affiliations:** 1 Hypertension Unit, University of Ottawa Heart Institute, 40 Ruskin Street, Ottawa, ON K1Y 4W7, Canada; 2 Laboratory of Genetics of Cardiac Diseases, University of Ottawa Heart Institute, 40 Ruskin Street, Ottawa, ON K1Y 4W7, Canada

## Abstract

**Background:**

To test whether epithelial sodium channel (ENaC) genes' variants contribute to salt sensitive hypertension in Dahl rats, we screened ENaC α, β, and γ genes entire coding regions, intron-exon junctions, and the 3' and 5' flanking regions in Dahl S, R and Wistar rats using both Denaturing High Performance Liquid Chromatography (DHPLC) and sequencing.

**Results:**

Our analysis revealed no sequence variability in the three genes encoding ENaC in Dahl S *versus *R rats. One homozygous sequence variation predicted to result in a D75E substitution was identified in Dahl and Wistar rat ENaC α compared to Brown Norway. Six and two previously reported polymorphic sites in Brown Norway sequences were lost in Dahl and Wistar rats, respectively. In the 5' flanking regions, we found a deletion of 5GCTs in Dahl and Wistar rat ENaC α gene, five new polymorphic sites in ENaC β and γ genes, one homozygous sequence variation in Dahl and Wistar rat ENaC γ gene, as well as one Dahl rat specific homozygous insertion of -1118CCCCCA in ENaC γ gene. This insertion created additional binding sites for Sp1 and Oct-1. Five and three Brown Norway polymorphic sites were lost in Dahl and Wistar rats, respectively. No sequence variability in ENaC 3' flanking regions was identified in Dahl compared to Brown Norway rats.

**Conclusion:**

The first comprehensive sequence analysis of ENaC genes did not reveal any differences between Dahl S and R rats that were isogenic in the regions screened. Mutations in ENaC genes intronic sequence or in ENaC-regulatory genes might possibly account for increased ENaC activity in Dahl S *versus *R rats.

## Background

The epithelial sodium channel (ENaC) is made up of three homologous subunits named α, β, and γ that assemble together to form a highly Na^+^- selective channel [1]. The structure of these subunits is characterized by the presence of two transmembrane domains separated by a large extracellular loop. Identification of mutations in ENaC subunits causing salt-sensitive hypertension and hypotension in humans (Liddle's syndrome and pseudohypoaldosteronism type 1) highlighted the impact of these genes on salt homeostasis and control of blood pressure (BP) [2,3].

In rats, the three α, β and γ ENaC (rENaC) subunits are encoded by three distinct genes *Scnn1a, Scnn1b*, and *Scnn1g *respectively, located on chromosomes 4q42, 1q36-41 and 1q36-41. The three subunits share similar structures and show 33–37% amino acid sequence homology in human [4]. rENaC α gene is composed of 12 coding exons [5]. rENaC β and γ genes are composed of 13 exons, the translation initiation codon is present within the second exon for both genes [6,7].

Dahl rats represent a robust animal model of genetically determined salt-sensitive hypertension. High salt intake increases BP in Dahl salt-sensitive (Dahl S), but not in Dahl salt-resistant (Dahl R) rats. Blockade of ENaC in the brain by benzamil prevents the increase in BP in Dahl S rats on high salt diet [8].

So far, only the coding sequences of genes encoding the three subunits have been partially screened in Dahl S and R rats. Analysis of near full length (base 22 till the end, all numbering starts at the A nucleotide of the primary initiation codon) of the *Scnn1b *cDNAs derived from kidneys of Dahl S and R rats failed to reveal any coding sequence mutations that could affect the predicted peptide sequence of *Scnn1b *[9]. Sequencing of nucleotides 21667 to 22054, 31172 to 31492, and 29142 to 29522 in the carboxy termini of ENaC α, β, and γ genes respectively revealed no differences in Dahl S *versus *R rats [10]. The sequence of the entire coding regions, intron-exon junctions, as well as the 3' and 5' flanking regions of ENaC three genes has not yet been reported in Dahl rats. In order to identify any variation, each sequence was analyzed using a combination of two screening methods, Denaturing High Performance Liquid Chromatography (DHPLC), offering 95–100% sensitivity and 100% specificity and automatic sequencing, offering 99.7–100% sensitivity and 100% specificity [11,12]. Dahl S and R rats, as well as Wistar rats, used as a control, were screened and the obtained sequences were compared to Brown Norway sequences retrieved from the rat genome database.

## Results

### Screening of ENaC α, β, and γ Genes Coding Regions for Variations between Dahl R and S rats

No sequence variability was identified in the entire coding regions, as well as in exon-intron junctions of ENaC α, β, and γ genes in Dahl S *versus *R rats (Table 1). One homozygous sequence variation G225T in exon 1 of ENaC α, predicted to result in a D75E substitution, was identified in Dahl S and R and Wistar rats compared to the published Brown Norway sequence (Table 1). Previously published Wistar rat sequences did not identify this variation [5]. It is therefore possible that Wistar rats are heterozygous at this position. One reported Brown Norway polymorphic site of ENaC α was lost in both Dahl and Wistar rats (Table 1). No sequence variability was identified in Dahl and Wistar rat ENaCβ gene compared to the Brown Norway sequence available in the public domain. No sequence variation in ENaC γ gene was identified in Dahl and Wistar rats compared to the published Brown Norway sequence. However, five and one previously reported polymorphic sites in Brown Norway ENaC γ sequence were lost in Dahl and Wistar rats, respectively (Table 1). The Wistar rat alleles studied followed a Mendelian independent assortment.

**Table 1:** Allelic variants in the coding sequence of ENaC subunits in Dahl S, R, Wistar, and Brown Norway rats

Subunit	Nucleotide position/exon	Dahl S and R	Wistar	Brown Norway	AA change
**ENaC α**	+225/1	TT	TT*	GG	D75E
	+10716/3	AA	AA	GG, GA, AA	R290R
**ENaC γ**	+4008/4	GG	GG	AA, AG, GG	E255K
	+22549/7	CC	TT, TC, CC	TT, TC, CC	D376D
	+24672/8	CC	TT, TC, CC	TT, TC, CC	C410C
	+29195/13	TT	CC, CT, TT	CC, CT, TT	C542C
	+29291/13	TT	GG, GT, TT	GG, GT, TT	C573W

Numbering starts at the A nucleotide of the primary initiation codon [15].

AA: amino acid

* GG genotype was previously reported [5].

### Screening of ENaC α, β, and γ Genes 3' and 5' Flanking Regions for Variations between Dahl R and S rats

**Figure 1:**
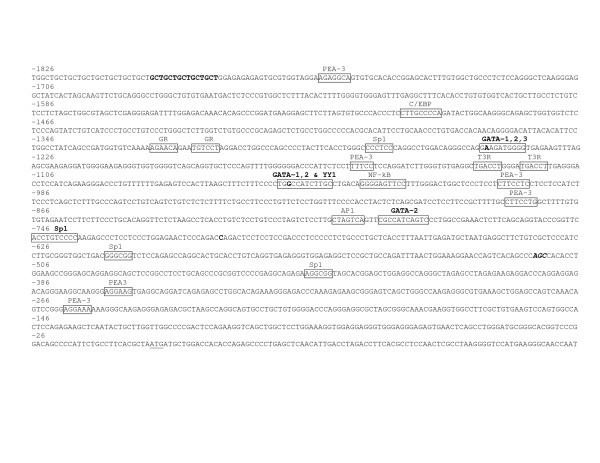
Location of the variants identified in the 5' flanking region of Dahl S, R, and Wistar rats ENaC α gene on the Brown Norway rat genomic sequence [15]. Position of the variants identified in the current study is highlighted in bold. Boxes represent the putative transcription factor-binding sequences; the putative binding sequences found during the present sequence analysis are labeled in bold; the factor names are written above the boxes. The first three bases for the major kidney and brain transcription start sites are italicized and bold. The translation initiation codon (+1) is underlined. TFSEARCH^® ^scores for the newly assigned putative binding sequences are 93.1, 89.7, and 89.7 for GATA 1, 2, 3 respectively; 89.0 and 88.5 for GATA 1, 2 respectively and 85.8 for YY1; 88.5 for GATA 2, and 87.7 for Sp1.

**Figure 2:**
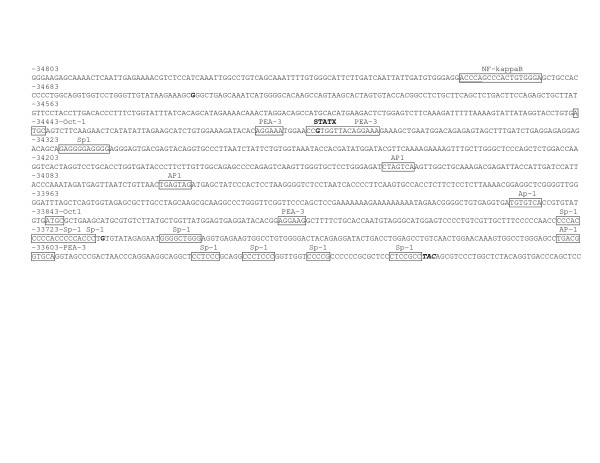
Location of the variants identified in the 5' flanking region of Dahl S, R, and Wistar rats ENaC β gene on the Brown Norway rat genomic sequence [15]. Position of the variants identified in the current study is highlighted in bold. Boxes represent the putative transcription factor-binding sequences; the putative binding sequences found during the present sequence analysis are labeled in bold; the factor names are written above the boxes. The first three bases for the major kidney and brain transcription start sites are italicized and bold. TFSEARCH^® ^score for the newly assigned putative binding sequence for STATX is 92.3.

**Figure 3:**
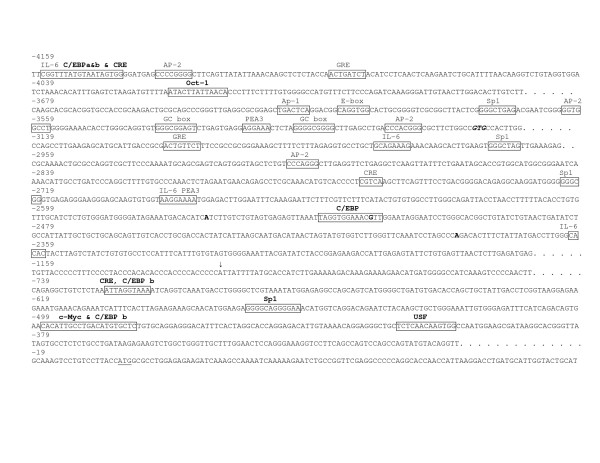
Location of the variants identified in the 5' flanking region of Dahl S, R, and Wistar rats ENaC γ gene on the Brown Norway rat genomic sequence [15]. Position of the variants identified in the current study is highlighted in bold. Boxes represent the putative transcription factor-binding sequences; the putative binding sequences found during the present sequence analysis are labeled in bold; the factor names are written above the boxes. The first three bases for the major kidney and brain transcription start sites are italicized and bold. The translation initiation codon (+1) is underlined. TFSEARCH^® ^scores for the newly assigned putative binding sequences are 89.2, 87.4, and 89.0 for C/EBP a & b and CRE, respectively; 85.8 for Oct-1; 89.3 for C/EBP, 87.9 and 85.5 for CRE and C/EBPb respectively; 87.7 for Sp1, 91.0 and 87.4 for c-Myc and C/EBPb respectively; and 85.9 for USF.

We first defined the 5' flanking regions to be screened for each ENaC subunit. The predicted putative binding sites on Brown Norway rat ENaC sequences were identical using TRANSFAC^® ^and TFSEARCH^®^. Because of the presence of potential kidney and brain transcription factor binding sites, we screened 1.8 kbp, 1.5 kbp, and 4.4 kbp of the 5' flanking regions of ENaC α, β, and γ respectively from the transcription start site (Figures 1, 2, and 3). Compared to previously reported transcription factors binding sites [5-7,13], the present analysis determined five, one, and ten new potential transcription factor binding-sites sequences on ENaC α, β, and γ, respectively (Figures 1, 2, and 3).

No sequence variability was identified in both the 3' and 5' flanking regions of ENaC α, β, and γ genes in Dahl S *versus *R rats (Table 2).

**Table 2:** Allelic variants in the 5' flanking regions of ENaC subunits in Dahl S, R, Wistar, and Brown Norway rats.

**Subunit**	**Nucleotide Position**	**Dahl S and R**	**Wistar**	**Brown Norway**
**ENaC α**	-705	TT	TT	CC, CT, TT
	-1050	AA	AA	GG, GA, AA
	-1247	TT	TT	AA, AT, TT
	-1788	Deletion of 5GCTs	Deletion of 5GCTs	
**ENaC β**	-34381	AA	GG, GA, AA	GG, GA, AA
	-34648	AA	GG, GA, AA	GG
**ENaC γ**	-1118	Insertion of CCCCCA		
	-1588	CC	AA, AC, CC	AA
	-2054	TT	TT, CT, CC	CC, CT, TT
	-2386	GG	AA*	AA
	-2525	AA	GG, GA, AA	GG
	-2561	GG	AA, AG, GG	AA
	-3313	CC	CC	TT

Numbering starts at the A nucleotide of the primary initiation codon [15].

* GG genotype was previously reported [7].

Four homozygous sequence differences were found in both Dahl and Wistar rat ENaC α gene compared to Brown Norway: one homozygous 15 bp deletion (-1788 → -1803 bp), and the loss of three Brown Norway polymorphic sites (Table 2). In addition to previously described transcription factor-binding sites [5,13], computer analyses suggest that the regions from -1248 to -1239, from -1052 to -1047, from -780 to -790, and from -736 to -746 represent putative binding sites for GATA-1, -2, and -3, GATA-1 and 2 and YY1, GATA-2, and Sp1 respectively (Figure 1). The putative GATA-1, -2, and -3 binding site (-1248 to -1239) found in presence of the -1247A allele loses the binding site for GATA-2 in the presence of the T allele. The putative binding site for YY1 (-1052 to -1047) found in presence of the -1050G allele and overlapping GATA-1 and 2 sites, is lost in the presence of the A allele; while GATA-1 and 2 transcription binding sites remained unaltered. Both Dahl and Wistar rats were homozygous TT and AA for respectively the T-1247A and the G-1050A polymorphisms.

The G-34648A ENaC β gene variant, not reported in Brown Norway rats, was present at the homozygous state in Dahl rats and at the heterozygous state in Wistar rats (Table 2). One Brown Norway ENaCβ gene polymorphic site was lost in Dahl rats, but not Wistar rats (Table 2). In addition to previously described transcription factor-binding sites [6] the regions from -34650 to -34635 represent a putative binding site for STAT proteins respectively (Figure 2). However, the presence of the A-34648G polymorphism within this STATX potential site is not predicted to alter STAT protein binding. Dahl rats were homozygous AA for the G-34648A polymorphisms, while Wistar rats were heterozygous.

Four ENaC γ gene sequence variations (A-1588C, G-2525A, A-2561G and T-3313C) were identified in both Dahl and Wistar rats compared to Brown Norway rats, these variations were all homozygous in Dahl rats; in Wistar rats T-3313C was found at the homozygous state, while A-1588C, G-2525A, A-2561G were heterozygous (Table 2). A 6 bp deletion at -1118 was only found in Dahl rat ENaC γ gene (Table 2). The A-2386G ENaC γ gene variant, not reported in Brown Norway rats, was present at the homozygous state in Dahl rats. Our screening of the Wistar ENaC γ gene only found the homozygous AA allele, however, the presence of the G allele was previously reported [7] (Table 2). One previously reported Brown Norway polymorphic site ENaC γ was lost in Dahl rats, but not Wistar rats (Table 2). In addition to previously described transcription factor-binding sites [7], the regions from -4102 to -4120, from -4010 to -4022, from -2483 to -2497, from -696 to -706, from -607 to -619, from -442 to -463, and from -400 to -413 represent putative binding sites for C/EBP a & b and CRE, Oct-1, C/EBP, CRE and C/EBP b, Sp1, c-Myc and C/EBP b, and USF respectively (Figure 3). One polymorphism, the G-2525A polymorphism, out of the seven found in the 5' flanking region of ENaC γ gene, is located within a potential consensus binding sites for C/EBP (Figure 3). The C/EBP binding site is present when the -2525G allele is present and the binding site is lost in the presence of the A allele. Dahl rats were homozygous AA for the G-2525A polymorphism, while Wistar rats were heterozygous and Brown Norway rats homozygous for the G allele. Finally, the 6 bp homozygous insertion at position -1118, which is only present in Dahl rats, is predicted to create Sp1 and Oct-1 sites (CCCCA*CCCCA*TT) (TFSEARCH^® ^scores 87.7 and 85.8, respectively).

## Discussion

The results of the current study show that Dahl S and R inbred rats are isogenic in the entire coding regions, exon-intron junctions, 3' and 5' flanking studied regions of ENaC α, β, and γ genes. These results are in agreement with the initial partial screenings of ENaC subunits in Dahl rats [9,10]. Nine homozygous sequence variations were identified in ENaC genes in Dahl rats compared to the Brown Norway sequence available in the public domain and eight of these nine sequence variations (5 polymorphic and 3 homozygous) were also identified in Wistar rats. However, the 6 bp deletion at -1118 which in the γ ENaC 5' flanking region, was specific for Dahl rats and not found in Wistar or Brown Norway rats. Eleven and five Brown Norway polymorphic sites were lost in Dahl and Wistar rats respectively. Among the identified variations in Dahl and Wistar rats, three are non synonymous variations. Four variants in the 5' flanking regions of ENaC α, β, and γ genes are present within putative DNA consensus regulatory elements and two putative DNA consensus sites were introduced with the Dahl rat specific 6 bp insertion at -1118 in ENaC γ.

Of the three non synonymous variants identified in the present study (αD75E, γE255K, and γC573W), only γC573W, located in the transmembrane domain M2, was previously functionally assessed and was found not to modify ENaC activity in *Xenopus *oocytes [10]. The αD75E substitution is present in the N terminus of the channel, prior to the transmembrane domain M1 and close to the channel pore, a critical region for kinetics properties of the channel predicted to participate in channel gating [10,14]. γE255K located in the extracellular loop might alter the amiloride sensitivity since residues essential for the formation of the high affinity amiloride-binding sites reside within this domain [1]. The silent polymorphism C542C was previously documented in ENaC γ carboxy terminus within and between rat strains [10].

Previous studies analyzed 1.5 kbp of ENaC α, 1.3 kbp of ENaC β, and 4.2 kbp of ENaC γ from the transcription start site in Wistar, Sprague Dawley and Wistar rats respectively as well as the first intron in Wistar rat ENaC γ [5-7,13]. In the present analysis, we determined five, one, and ten new potential transcription factor binding-sites sequences on ENaC α, β, and γ, respectively. Among all variants identified within putative transcription factors binding sites, one of them, the insertion of -1118CCCCCA, located on ENaC γ gene was only present in Dahl rats. To our knowledge, this variant has not been previously reported on Dahl rats or any other rat strains [7,15,16]. It creates binding sites for Sp1 and Oct-1. Sp1 is a DNA-binding protein which interacts with a variety of gene promoters containing GC-box elements. Moreover, the activity of TATA-less promoters is frequently dependent on Sp1 sites in the proximal promoter region. Deletion of one of the two clusters of Sp1 consensus binding sites within the 5' flanking region of the ENaC β gene indicated that the proximal cluster was essential to basal promoter activity in transfected cell lines [6]. Studies of both the human and rat γ -subunits [7,17] also reported the absence of a TATA box in their promoters and the presence of GC boxes and Sp1 consensus sites. In human ENac γ, Sp1 may be part of the transcription complex that binds at the core promoter [17]. Sp1 protein is part of a much larger family of mammalian transcription factors, the Sp/XKLF family [18], it is ubiquitously expressed, and contains three highly conserved C2H2-type zinc fingers in the C-terminal region and have a glutamine-rich activation domain. Sp1 can bind GC boxes, and acts as a transcriptional activator. Therefore the presence of a potential additional Sp1 consensus site in Dahl rats may enhance the transcription of the ENaC γ gene in Dahl rats compared to other rat strains. Similarly, Oct-1 sites have previously been reported to be able to function as repressors or activators of transcription depending on context [19-21]. Overexpression of ENaC γ in collecting duct cells has been shown to enhance Na^+ ^transport [22]. Remarkably, overexpression of marginally detectable amount of ENaC γ was sufficient to produce a full increase in Na^+ ^transport [22]. However, determination of the precise mechanism of all above variants in influencing promoter activity awaits further investigation.

It remains possible that mutations in the intronic regions of the ENaC are involved in the generation of hypertension in Dahl S rats on high salt intake. Commonly occurring ENaC variants, including intronic substitution (i12-17CT) were found associated with an increased urinary potassium excretion rate in relation to the renin levels as well as with hypertension in humans [23]. However, when expressed in *Xenopus *oocytes, the variants did not show a significant difference in activity compared with ENaC wild-type [23].

One can speculate that mutations in genes encoding a protein interacting with ENaC to regulate its activity might increase ENaC activity leading to hypertension. SGK1, which activates ENaC in tubules, maps to a known BP QTL [24]. Abnormal regulation of SGK1 mRNA and protein level by aldosterone in Dahl S compared to Dahl R rat was observed suggesting that regulation of ENaC *via *SGK1 signaling pathway may be disturbed in Dahl S rat [25].

## Conclusion

To our knowledge, this report presents the first comprehensive screening for variations in the entire coding sequences including intron-exon junctions, and in the 3' and 5' flanking regions of ENaC three genes in the hypertensive Dahl S rats and their normotensive Dahl R control rats together with an additional control group of Wistar rats. We could not link salt induced hypertension in Dahl S to differences in ENaC sequences in Dahl S *versus *R rats. Further characterization of SNPs across candidate genes contributing to the salt-sensitive hypertension phenotype will be useful in designing genetic mapping panels for association studies. If disordered activity of the epithelial cell sodium channel contributes to the pathogenesis of hypertension in Dahl S rats, it appears to stem from genetic variations in genes encoding proteins that regulate ENaC or in intronic sequences important for the structure or function of the sodium channel.

## Methods

### Animals

Male Dahl S and R rats (4 rats/group), 4–5 wks of age, were obtained from Harlan Sprague Dawley (Indianapolis, IN) and handled as previously described [8]. To assess the salt sensitivity of Dahl S rats, at 5 wks of age, Dahl S and R rats were placed on a high-salt (1,370 μmol Na/g, Teklad; Madison, WI) diet for 4 wks. After 4 wks, BP was measured invasively by intraarterial catheter and the average mean arterial pressure was estimated to be 156 ± 11 for S, and 131 ± 1 for R rats (P < 0.05). Wistar rats (Charles River Breeding Laboratories Montreal, QC, Canada) were used as control, and were not subjected to high salt diet. The animals were then killed by decapitation and whole blood was collected for DNA isolation. All experiments were carried out in accordance with the guidelines of the University of Ottawa Animal Care Committee for the care and use of laboratory animals.

### Genomic DNA isolation and amplification

Genomic DNA was isolated from white blood cells (Qiagen FlexiGene, Qiagen Canada, Mississauga, ON, Canada). Using PRIMER3-based Web application [26], 73 sets of specific oligonucleotide primers (Table 3) where designed based on the November 2004 rat (*Rattus norvegicus*) genome assembly [15] in order to screen the coding sequence of ENaC three genes, including the exon-intron boundaries, as well as the 5' and 3' flanking regions [GenBank: NM_031548; NM_012648; NM_017046] [GeneID: 25122; 24767;24768]. For large exons (> 350 bp) overlapping primer sets were employed.

**Table 3A:** Primers designed to screen the coding sequence of ENaC α, β, and γ genes. Optimum temperatures (°C) for PCR and DHPLC are included in the table.

		Sequence	PCR	DHPLC
**ENaC α**				
Exon 1	Forward	GGTAGCACGGAGCTGGAG	64	63
	Reverse	CCCAGGCTGAGTTCACTCTC		
	Forward	AGCTCAATACTGCTTGGTTGG	61	63
	Reverse	GAAGAGCTCCCGGTAGGAG		
	Forward	GGGACAAACGTGAAGAGCAG	61	63
	Reverse	CTTGCTTTTGTGCTGCTGAG		
Exon 2	Forward	CACTGATCCCCTCCGTGTTA	60	64
	Reverse	TCCATCAGGCCTCTATCTGAA		
Exon 3	Forward	GCTCTCTGTCCCTCACCTTG	63	62
	Reverse	ACCACCAAGCATTTCCTGAG		
Exon 4	Forward	CACATTGGAGGTGACAGGAA	63	61
	Reverse	CCAAGGTGAGACCCAGAAGA		
Exon 5	Forward	GCTGGCCTTGTTCTATCAGG	62	62
	Reverse	CTCCCTTCAGTCTCCTGCTG		
Exon 6	Forward	GGTGAAGCCTGAGTCATTCC	63	62
	Reverse	CCAACCTACTTCCCCTCCAT		
Exon 7	Forward	GAGGGATGGAGGGGAAGTAG	61	61
	Reverse	AGCCAGCACCTAGGGAAAA		
Exon 8	Forward	GGCACCATTGAAATGCTCTT	59	61
	Reverse	ATCAAAGTGCCCAGTTACGG		
Exon 9	Forward	GCAGCTGCTTAACCTGGTAGA	61	61
	Reverse	ATGTCCACTTGTGCGTGTGT		
Exon 10	Forward	CCATCCCTGTAAACATGAGG	61	61
	Reverse	CCCCAATATCTCCACCAGAA		
Exon 11	Forward	TGGTGGAGATATTGGGGAGA	61	61
	Reverse	CAAACCCTTCTGACCCTTCA		
Exon 12	Forward	TGACAGGAGGCGCTAGAGT	62	63
	Reverse	AGTAGCATAGGCAGGTGGAG		
	Forward	CTCCACTCCAGCTTCCTCCT	62	63
	Reverse	ATCGTTAGCCCCTGTCCTCT		
	Forward	TCTCACTTCAGCACATCTTCC	61	63
	Reverse	GGCCTACCCTGGTCTGTCTT		
	Forward	ACCCAAAAGCCCCCTTGT	61	61
	Reverse	AGTACACTGTGGGGGTGAGG		
	Forward	CCAAAGGCACCATTTCTTTT	59	61
	Reverse	ATGTAGGCGGTGCCTCAG		
**ENaC β**				
Exon 2	Forward	CTAGTCTCCAGGCCCATGAC	62	64
	Reverse	CTACTGGAAGGGGCTGGAAT		
Exon 3	Forward	CCCCATGTTCCACACTCTTT	60	62
	Reverse	AACAAAAATCGATTGCTACCAG		
Exon 4	Forward	TAATACGGTGCTGCCATTCC	61	63
	Reverse	GCATAGATCAGCCTGTGTGC		
Exon 5	Forward	CTCCAGCAGAGCAGGACAAT	62	61
	Reverse	GGTCTTTCCGCCCTGTGT		
Exon 6	Forward	GGTGATGGCCTCCCTTCTAT	61	61.5
	Reverse	AGGCAGCCTGAACACAAGAG		
Exon 7	Forward	GCTCCATGGGGAGGTACATA	63	63
	Reverse	GTCCGGCCCTCATAGGTAAG		
Exon 8	Forward	AAAGTCTCTGGGCTCCAAGG	63	61.5
	Reverse	AGAGGCCCCCTTGCCTAAC		
Exon 9	Forward	GAGGGAAGACCCCTGGAAG	62	63.5
	Reverse	ATACTGGGTGTCGCTGGAAG		
Exon 10	Forward	TCCTGCAAGTGAGTGTGTCC	62	63
	Reverse	AGGGGGAGAAGACCCTCTTT		
Exon 11	Forward	AGCATGTGTGTGCGTGTGTA	60	63
	Reverse	CAGCAAGAGCAGTTTGGACA		
Exon 12	Forward	CAACTCAGACTGGAGATGAGCA	62	62.5
	Reverse	GGAACCATAACCCCCACCTA		
Exon 13	Forward	CCTAGGTGGGGGTTATGGTT	62	64
	Reverse	GCAGCCTCAGGGAGTCATAG		
	Forward	CTTCCAGCCTGACACAACC	62	63.5
	Reverse	GCCTGTCTGCTAGGTCAACA		
	Forward	CTGAGGGGTTCATAGGGTCA	62	60.5
	Reverse	ATCCTACACCCCAGACATGC		
**ENaC γ**				
Exon 2	Forward	AGTCGCAGGCTCCAGAGAT	62	64
	Reverse	GAGGGGCTTTGACATCCAT		
Exon 3	Forward	GGAAGGCAACATGAAGAAGC	61	60
	Reverse	ACTGTGAGCCCACCAGCTC		
Exon 4	Forward	AAGGACCTACCCTGGCATCT	63	60
	Reverse	CTCAAGGCCTACAGGTGAGC		
Exon 5	Forward	ACGCAATGCTTCCTGACTTC	60	60
	Reverse	TGCACTGCTGCTGTCAAGAT		
Exon 6	Forward	CCCTGGGGACTGCTTTTT	60	60
	Reverse	TCCATTAGCAGCACCTCCTT		
Exon 7	Forward	AGGGGAATCCTCCTATCTGG	61	62
	Reverse	CCTTGGCCTAGATATAGCTTCA		
Exon 8	Forward	AGGGAGTTCCCGGTCTCTAC	63	61
	Reverse	GCGTGGCCAAGCTGATTC		
Exon 9	Forward	AGATGGTGGAGGTTCCACAG	63	60
	Reverse	GGGAGAAAGGCACAGAGTGA		
Exon 10	Forward	ACTGGGGCAGGTAGGACTTT	62	60
	Reverse	GCTTTGGCTGTGTTGCTGTA		
Exon 11	Forward	CCAAAGCCAGAGACAGGTTG	59	61
	Reverse	TCGAATGAACGAAAAGGTGA		
Exon 12	Forward	ACCTGGCAGGAAGCCAAG	62	61
	Reverse	CCCTCTGGCAGCAAAACTAC		
Exon 13	Forward	CCCTGAGTGCAGGATTTATCA	61	64
	Reverse	GTATCTGGGAGGTGGTGTGC		
	Forward	GTCAGTGGCACAAAGCCAAG	62	64
	Reverse	AGCTCATAAGTGCCAAGTCCA		
	Forward	CCTGCTGTGAACCGGATA	60	60
	Reverse	GGCCAACTGTCTGTCTGAGG		
	Forward	GCCAGCTATTGCCTGACAT	60	60
	Reverse	CGCATACTCTCAGTTCAAAGACA		
	Forward	GCCAAATGGTATTCCCACAA	59	57
	Reverse	ATTGGACTAGCCTGGGTGCT		

**Table 3B:** Primers designed to screen the 5'flanking sequence of ENaC α, β, and γ genes. Optimum temperatures (°C) for PCR and DHPLC are included in the table.

		Sequence	PCR	DHPLC
**ENaC α**				
	Forward	CAGATTCAGCTGCCATGC	60	62
	Reverse	AGGCCCTGCAGAACTTGCT		
	Forward	GGTAGGAAGAGGCAGTGTGC	61	62
	Reverse	ACAGGGTTGCAGGAATGTG		
	Forward	GCTGGTGGTCTCTCCCAGTA	64	62
	Reverse	GGTCCCTTCTGATGGAGGTC		
	Forward	GGGGGACCCATTCTCCTT	62	59
	Reverse	GGACTGATGGCGAACTGACT		
	Forward	CCTTCCTGGCTTTTGTGTGT	61	62
	Reverse	GTGTGGCTGGGCTGTGAC		
	Forward	CAGGCACTGCACCTGTCA	62	62
	Reverse	GGCTTAGCGTCTCTCCCTCT		
	Forward	CACAGAAAGGGAGACCCAAA	60	63
	Reverse	GGTCTTGCTCCTTGAATTGG		
	Forward	GAGGGCAGCCTGGGATGCGG	61	62
	Reverse	TTATAATAGCAATAGCCCCA		
	Forward	TCCAAGGAGAAGGCGCCCCCA	61	62
	Reverse	AGGGCTGGGTGAGAGGAT		
	Forward	GGTTTAAGGATTTGCTTGATTC	61	61
	Reverse	TGTTCTGCAAGGACAGCATC		
	Forward	CAAAGTACCCAATATCTATT	61	61
	Reverse	AGGGCTGGGTGAGAGGAT		
	Forward	CCTGGTTTTGGGGTGTGT	61	61
	Reverse	TGTTCTGCAAGGACAGCATC		
	Forward	GCTCTCTTTGGGCTGTGGGGAC	61	61
	Reverse	TGTTCTGCAAGGACAGCATC		
**ENaC β**				
	Forward	GGTCTTCTGGGAAGAGCAAA	61	62
	Reverse	CACGGTTCCATTTCCTGTGT		
	Forward	CAGCCATGCACATGAAGACT	61	57
	Reverse	TTGCAGCCAACTTGACTAGAT		
	Forward	TGGTGATACCCTTCTTGTTGG	61	60
	Reverse	CCTCCGTGTATCCTCACTCC		
	Forward	AAAATAGAACGGGGCTGTGA	61	62
	Reverse	GGAGCTGGGTCACCTGTAGA		
	Forward	TGACCTGGAGCCTGTCAACT	62	64
	Reverse	GACGGAACTGCGGTCATT		
**ENaC γ**				
	Forward	CTTGACATGTTTCTACCCACCA	61	58
	Reverse	TTAGAACGCTGAAACCGTGA		
	Forward	CATCCTCAACTCAAGAATCTGC	60	60
	Reverse	CCACTCTGCAAGCTGCATTA		
	Forward	TGCAGAAGCAGCAGTAAGAGA	61	62
	Reverse	CTGGCGTGTGTACAGTCTGG		
	Forward	CTTCTGGCCGTGCCACTT	61	62
	Reverse	TCGGGCTCCTCTTTCAACTA		
	Forward	CGCGGGAAAGCTTTTCTTTA	61	63
	Reverse	CTTGCTCCCTTCCCTCTCAC		
	Forward	ATGAACAGAGCCTCGCAAAC	61	62
	Reverse	GATAGTGGTCGCAGGTGACA		
	Forward	GGTGGAAACGTTGGAATAGG	62	58
	Reverse	AAAGCAAGGCTGTCGCTCTA		

The nucleotides representing the entire coding sequences and the 3'UTR and flanking regions that were screened in ENaC genes are as follows (numbering starts at the A nucleotide of the primary initiation codon): for ENaC α, nt. 1 to 590, 9199 to 9652, 10553 to 10887, 15934 to 16281, 16442 to 16777, 17027 to 17271, 17246 to 17536, 20442 to 20730, 20663 to 20874, 20927 to 21134, 21117 to 21372, and 21582 to 23041; for ENaC β, nt. 1 to 413, 3287 to 3686, 5622 to 6012, 23724 to 23969, 25539 to 25839, 25974 to 26213, 27914 to 28154, 28987 to 29221, 29116 to 29351, 29910 to 30157, 30857 to 31100, and 31078 to 32049; for ENaC-γ nt, 1 to 378, 2108 to 2552, 3781 to 4141, 5153 to 5403, 8093 to 8385, 22437 to 22674, 24560 to 24797, 25439 to 25676, 25616 to 25849, 25842 to 26091, 28671 to 28918, 29065 to 30547. As for the 5' flanking regions of ENaC genes, -2078 to +1 bp, -34812 to -33582 bp, -4359 to +1 bp of ENaC α, β, and γ genes respectively were amplified.

### Sequence Analysis

a) DHPLC (Helix, Varian, Palo Alto, CA) analysis. DHPLC runs were performed as recommended by Varian using buffer A and buffer B (Varian) and a flow rate of 0.45 ml/min. Freshly prepared PCR products were denatured at 95°C for 3 min and re-annealed by decreasing the temperature from 95°C to 64°C at a rate of 1°C/min. Optimal melting temperatures for the PCR products were determined using the Stanford University website [27]. The chosen temperatures correspond to the point at which the retention time was 75% of (*t*_rmax _-*t*_rmin_). b) Automatic sequencing (ABI 310, PE Applied Biosystems, Foster City, CA). Sequencing was performed using the DYEnamic ET Terminator kit according to the instructions provided by the manufacturer (PE Applied Biosystems, Foster City, CA). Sequencing products were purified (DyeEx 2.0 spin kit columns; Qiagen Canada, Mississauga, ON, Canada) and analysed on 310 DNA analyser (Applied Biosystems, Foster City, CA). Resulting sequences were compared to the Brown Norway sequence [15]. c) ENaC 5' flanking regions analysis. A comprehensive analysis of putative kidney or brain transcription factor binding sites was performed using both literature reports [6,7,13] and two well-known and large-scale databases, TRANSFAC [28]^® ^ and TFSEARCH^® ^[29]. Using the cell selectivity track, the database searches were refined to transcription factors active in the rat kidney and brain where ENaC contributes to BP control.

## Authors' contributions

MS performed the all experiments and sequences analysis, participated in experimental design, drafted the manuscript.

FL conceived the project, finalized the manuscript.

FT designed the experimental strategy, supervised the study, and finalized the manuscript.

All authors read and approved the final manuscript.
